# ADHD and Risk of Precocious Puberty: Considering the Impact of MPH

**DOI:** 10.3390/biomedicines12102304

**Published:** 2024-10-10

**Authors:** Yi-Chun Liu, Yin-To Liao, Vincent Chin-Hung Chen, Yi-Lung Chen

**Affiliations:** 1 Department of Psychiatry, Changhua Christian Children’s Hospital, Changhua 500010, Taiwan; purpplewhale@yahoo.com.tw; 2 Department of Psychiatry, Changhua Christian Hospital, Changhua 500209, Taiwan; 3 Department of Healthcare Administration, Asia University, Taichung 413305, Taiwan; 4 Department of Psychiatry, China Medical University, Taichung 404333, Taiwan; je2tezy@yahoo.com.tw; 5 Department of Psychiatry, China Medical University Hospital, Taichung 404333, Taiwan; 6 School of Medicine, Chang Gung University, Taoyuan 33302, Taiwan; cch1966@gmail.com; 7 Department of Psychiatry, Chiayi Chang Gung Memorial Hospital, Chiayi 61363, Taiwan; 8 Department of Psychology, Asia University, Taichung 413305, Taiwan

**Keywords:** attention-deficit/hyperactivity disorder (ADHD), intellectual disability, methylphenidate (MPH), precocious puberty (PP), tics

## Abstract

Background/Objectives: The complex association between attention-deficit/hyperactivity disorder (ADHD) and methylphenidate (MPH) with precocious puberty (PP) is still unclear. This study aims to investigate the association between ADHD, MPH, and PP. Methods: This is a nationwide cohort study including a total of 3,342,077 individuals, 186,681 with ADHD and 3,155,396 without. First, we compared the risk of PP between ADHD cases and non-ADHD cases. Second, we compared the risk of PP between MPH users and non-MPH users in patients with ADHD. Results: Patients with ADHD were at a greater risk of PP (adjusted hazard ratio [aHR], 2.01 [95% CI, 1.91–2.11]). In our moderation analyses, the female gender was a positive additive effect modifier of the association between ADHD and PP, whereas tics and intellectual disability were negative effect modifiers. In patients with ADHD, MPH users had a significantly lower risk of PP (aHR, 0.63 [95% CI 0.57–0.70]), and females had a negative effect modification on the association between MPH and PP. Conclusions: Our study found that children with ADHD were at a greater risk of PP. Girls with ADHD were a group particularly vulnerable to PP. Comorbid tics or intellectual disability was associated with a lower risk of PP. Among patients with ADHD, MPH was protective against PP, especially in girls. However, these preliminary results need further validation due to the nature of them being from an electronic database study. Unmeasured confounding factors might affect the association between MPH and PP.

## 1. Introduction

Precocious puberty (PP) is diagnosed as the onset of secondary sex characteristics before age 8 in girls or age 9 in boys [1,2]. PP has a significant impact on the physical and psychological growth of children [1]. PP accelerates skeletal maturation and lead to early epiphyseal fusion, which may result in short stature in adulthood [1]. Furthermore, PP imposes a psychological burden on children, particularly on girls [1]. The rapid body changes associated with puberty can contribute to negative self-concepts regarding appearance in young children, potentially leading to feelings of shame or self-doubt that impair interpersonal relationships [3]. Research has indicated that children with PP exhibit higher levels of depression than those without PP, and girls are more likely to experience anxiety than boys [3]. The prevalence of PP varies by region and sex, ranging from 0.05 to 0.11% in boys and 0.2 to 4.11% in girls [4,5]. Girls are 4–38 times more likely to develop PP than boys [4,5]. The mechanisms contributing to the increased susceptibility of girls to PP are not fully understood. However, potential factors include hormonal influences, particularly estrogen’s role in growth velocity, genetic factors such as DLK1 (Delta Like Non-Canonical Notch Ligand 1) variants, and environmental changes, as evidenced by differing incidences of PP between sexes during the COVID-19 (Coronavirus disease 2019) pandemic [6]. Based on the source of sex hormones, PP can be categorized into central and peripheral types, of which the central type accounts for 80% of patients [7,8]. In most cases, the etiology of central PP remains idiopathic, and only in rare cases can it be associated with radiation exposure, congenital central nervous system abnormalities, specific genetic disorders, or hypothyroidism [9,10,11,12]. Identifying children at risk for PP will facilitate early intervention for the disease and improve their quality of life.

Attention-deficit/hyperactivity disorder (ADHD) is a neurodevelopment disorder characterized by inattention, impulsivity/hyperactivity, and executive dysfunction that affects 8–12% of children [13]. Boys are 3–4 times more likely than girls to be diagnosed with ADHD [14]. ADHD is a heterogeneous condition, with 60–80% of patients having at least one comorbid neuropsychiatric disorder [14,15]. Some of these comorbid neuropsychiatric disorders can be attributed to the complications of undiagnosed or untreated ADHD. However, some comorbid neuropsychiatric disorders, for example, autistic spectrum disorder, obsessive–compulsive disorder, intellectual disability, tics, anxiety disorder and epilepsy may share a common biologic basis with ADHD or have a unique and complex etiology that is distinct from ADHD alone [15,16,17,18,19]. Despite the heterogeneity of ADHD, psychostimulants are recognized as first-line medication [20]. In Taiwan, methylphenidate (MPH) is the only psychostimulant approved for the treatment of ADHD. MPH induces neuroadaptive changes, such as alterations in the density of dopamine transporters in the brain, changes in cortical variability, and modifications in brain network connectivity, and is thought to improve the core symptoms of ADHD [20,21,22].

Currently, very few studies have reported an association between ADHD and PP. Recently, one cohort study found that individuals with ADHD were about 1.5 times more likely to experience PP compared to those without ADHD, regardless of medication use (adjusted hazard ratio [aHR] = 1.511, confidence interval [CI]: 1.243–1.795) [2]. The study included 72,537 children with ADHD, of whom 66.3% were boys. ADHD in both females and males increased the risk of PP (hazard ratio [HR] = 1.37 and 1.53, respectively). However, in that study, the analysis of the medication was not comprehensive as it only included brand-name drugs and not commonly used generic ones. In addition, it did not account for the confounding and moderating effects of neuropsychiatric comorbidities, so the results should be interpreted with caution.

An association between MPH and puberty timing has been reported in animal studies. In female mice, MPH delays the onset of puberty [23,24,25], but in male mice, MPH accelerates the onset of puberty [24]. On the contrary, another study showed that MPH delayed puberty in male macaques [26]. The association between MPH and the timing of puberty remained uncertain [23,24,25,27]. Therefore, it is necessary to assess the effect of MPH on PP in patients with ADHD, while considering the moderating effect of sex.

We designed a population-based cohort study to investigate whether an association exists between ADHD and PP and, if so, whether MPH treatment alters this association. Given the heterogeneity of ADHD and the varied impact of MPH on pubertal timing between sexes, we also adopted moderation analyses of neuropsychiatric disorders and sex in our study.

## 2. Materials and Methods

### 2.1. Study Participants

**Figure 1:**
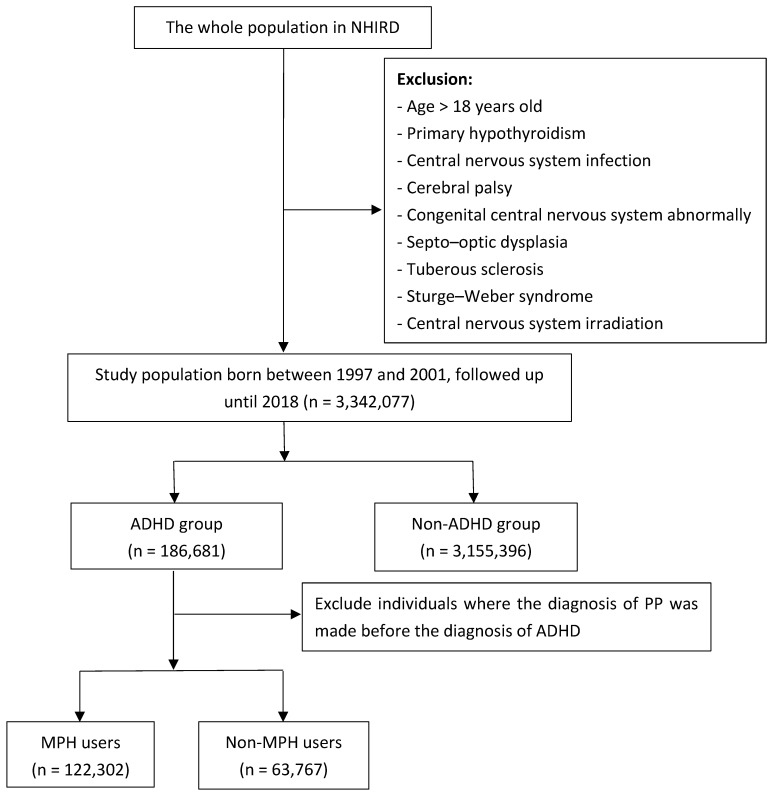
A flowchart of this study. Abbreviations: ADHD: attention-deficit/hyperactivity disorder; MPH: methylphenidate; n: number; NHIRD: the National Health Insurance Research Database; PP: precocious puberty.

All medical-claims data for National Health Insurance (NHI) beneficiaries were retrieved and de-identified to form Taiwan’s National Health Insurance Research Database (NHIRD). NHIRD, maintained and governed by the Health and Welfare Data Science Center of the Ministry of Health and Welfare, encompasses 99.6% of Taiwan’s total population [28]. We conducted a retrospective cohort study by using the full population dataset of the NHIRD from 1 January 1997 to 31 December 2018. Figure 1 presents a flowchart of our study design. Initially, we applied exclusion criteria to the entire population in the dataset. The study population was limited to those younger than 18 years old. Next, we retained individuals born between 1 January 1997 and 31 December 2010 from the remaining group, which constituted our study population. We followed them from their birth date to the index date of PP, death, or 31 December 2018, whichever came first. At the end of the follow-up period, the individuals in our study would be 8 years or older. Most cases of PP are diagnosed before or at this age [29].

We excluded individuals with certain organic factors that had been reported to cause PP [2,6], including the following: central nervous system infection, congenital abnormalities of the central nervous system, septo–optic dysplasia, Sturge–Weber syndrome, tuberous sclerosis, radiation exposure to the central nervous system, and primary hypothyroidism. The diagnosis codes we used were based on the International Classification of Diseases, Ninth Revision; Clinical Modification (ICD9-CM); and ICD-10 (Table S1 in Supplementary Materials).

### 2.2. Exposures

For evaluating the association between ADHD and PP, we set the exposure as ADHD. The corresponding ICD9-CM and ICD-10 codes for ADHD were 314 and F90, respectively. We identified ADHD cases if they had at least one medical record indicating ADHD as the primary diagnosis during the follow-up period. Most diagnoses of ADHD in the NHIRD are made by board-certified psychiatrists, which enhance diagnostic validity [30,31]. In addition, only medical records with confirmed diagnoses of diseases or disorders were classified as having those conditions, while all other records were categorized as not ill. This approach avoided the problem of missing data.

For evaluating the association between MPH and PP in patients with ADHD, we set the exposure as MPH prescription. Here, the study population would be limited to those who had been diagnosed with ADHD. The Anatomical Therapeutic Chemical (ATC) code we had adopted for MPH was N06BA04 (Table S2 in Supplementary Materials). The earliest date on which MPH was prescribed was defined as the index date of MPH prescription. In this way, each follow-up time for a given case may be divided into two different survival time intervals. Assuming that a patient with ADHD has been prescribed MPH during the follow-up period, and the period of MPH usage predates their diagnosis of PP or the end of this study, the time before the index date of MPH prescription would be defined as the patient’s non-MPH treatment period, while the time from the index date onward would be defined as the patient’s MPH treatment period. This approach ensures that the controls comprised person-time from individuals who had never used MPH before.

### 2.3. Outcomes

The main outcome is time-to-PP. The corresponding ICD9-CM and ICD-10 codes for PP were 259.1 and E30, respectively. Those who had at least one medical record of PP during the follow-up period were identified as cases with PP, and the earliest date on which PP was documented was defined as the index date of PP.

### 2.4. Effect Modifiers

Sex and neuropsychiatric comorbidities, namely, autistic spectrum disorder, obsessive–compulsive disorder, intellectual disability, tics, anxiety disorder, and epilepsy, were set as potential effect modifiers. We identified these comorbidities based on ICD systems (Table S3 in Supplementary Materials). Those who had at least one medical record of one of the specific comorbidities mentioned above during the follow-up period were identified as cases of that neuropsychiatric comorbidity.

### 2.5. Covariates

Neuropsychiatric comorbidities, sex, and low-income households were posited as covariates, provided that these covariates were not specified as effect modifiers. The definition of a low-income household is a family whose average monthly income of each family member is lower than the monthly minimum living expense of that residence region after the monthly family income is evenly distributed among the family members [32].

### 2.6. Statistical Analysis

We performed the statistical analyses by using SAS Version 9.4 (SAS Institute Inc., Cary, NC, USA). We used the Cox regression model to analyze survival data with age as the time scale. We conducted a preliminary analysis to assess the proportional hazards (PH) assumptions by using ln(−ln) plots and Schoenfeld’s residual tests. Only the sex variable did not satisfy the PH assumption; hence, we controlled for the sex variable by stratification.

The first part of the analysis focused on the association between ADHD and PP. We compared the association with PP between ADHD and non-ADHD groups with adjustment for low-income households, sex, and neuropsychiatric comorbidities. The Cox regression model was used to compute aHR for the risk of PP with the non-ADHD group as reference. Subsequently, we performed the stratification analyses and computed aHRs for each stratum based on sex. We also conducted moderation analyses of different neuropsychiatric comorbidities and sex based on the recommendations from the literature [33]. All variables were binary, and if sex was set as an effect modifier, we set female as “1” and male as “0”. We presented moderating effects on both additive and multiplicative scales. We adopted the relative excess risk due to interaction (RERI) to present additive effect modification and computed the 95% CIs based on the previous literature [34,35]. If the RERI is not equal to zero, it suggests that the association between ADHD and PP varies across strata defined by the effect modifier or that there is effect heterogeneity between strata [36]. A RERI greater than zero indicates a positive additive effect modification; that is, the effect of ADHD on PP was greater in the effect modifier groups (i.e., females or patients with specific neuropsychiatric comorbidities) than the non-effect modifier groups (i.e., males or patients without specific neuropsychiatric comorbidities). A similar principle can be explained when the RERI is less than zero. Regarding the multiplicative effect modification [37], if the value of the ratio HR_11_/HR_10_HR_01_ is not equal to one, it suggests that there is effect heterogeneity between strata defined by the effect modifier. A ratio greater than one indicates a positive multiplicative effect modification, that is, that the joint effect of exposure and the effect modifier exceeds the product of the effects of exposure and the effect modifier considered separately. A similar rationale can be used for interpretation when the ratio is less than one.

The second part of the analysis focused on the effect of MPH on PP. Initially, we compared the association with PP between MPH users and non-MPH users in patients with ADHD, adjusting for low-income households, sex, and neuropsychiatric comorbidities. We used the Cox regression model to calculate aHR for PP risk with the non-MPH users as reference. We also conducted stratification analyses to calculate the aHRs to compare differences in PP risk between the two groups within each sex-specific stratum. Then, we conducted stratification and moderation analyses of sex to examine whether the association between MPH and PP differed by sex.

## 3. Results

### 3.1. Demographic Characteristics of the Study Population

We initially enrolled 3,387,576 children born between 1997 and 2010, leaving 3,342,077 children after applying exclusion criteria. Of these, 186,681 were ADHD cases and 3,155,396 were non-ADHD cases. We conducted the descriptive statistical analysis, and Table 1 lists the demographic characteristics of the study population. After evaluating the skewness and kurtosis indicators, we found that the distribution of the variable age conformed to a normal distribution [38]. And the mean age at the end of the study period in the ADHD group was 14.8 years with a standard deviation of 3.8 years, while the mean age in the non-ADHD group was 15.2 years with a standard deviation of 4.0 years. Within the ADHD population, there were 146,006 male patients, accounting for 78.21%, and 40,675 female patients, accounting for 21.79%. The male-to-female ratio for ADHD was 3.59. Overall, 122,302 patients with ADHD had ever been treated with MPH, representing 65.51% of the population. Within the ADHD group, 2042 patients were diagnosed with PP, while in the non-ADHD group, 29,299 patients were diagnosed with PP. The incidence of PP was 1.09% and 0.93% in the ADHD and non-ADHD groups, respectively.

### 3.2. Risk of PP between ADHD Cases and Non-ADHD Cases

After adjusting for important confounders, the risk of PP was significantly higher in the ADHD group than in the non-ADHD group (aHR, 2.01 [95% CI, 1.91–2.11]) (Table 2). The results still hold in the stratification analysis of sex (males: aHR, 2.05 [95% CI, 1.86–2.26]; females: aHR, 1.99 [95% CI, 1.88–2.11]) (Table 2).

### 3.3. Moderation Analyses of Sex and Neuropsychiatric Comorbidities on the Association between ADHD and PP

Table 2 also lists the moderation analyses of sex and neuropsychiatric comorbidities. Females had a strong positive additive effect modification on the association between ADHD and PP (RERI, 8.56 [95% CI 7.48–9.64]). However, this moderating effect was not replicated on the multiplicative scale. Tics and intellectual disability had negative effect modifications on both additive and multiplicative scales; that is, the effect of ADHD on PP was attenuated in the presence of comorbid tics or intellectual disability. Autistic spectrum disorder and obsessive–compulsive disorder had negative effect modifications only on the multiplicative scales, but not on the additive scales.

### 3.4. Risk of PP between MPH Users and Non-MPH Users within Patients with ADHD and Moderation Analysis of Sex

Table 3 presents the effects of MPH on PP in patients with ADHD. Overall, in patients with ADHD, MPH users had a significantly lower risk of PP than non-MPH users (aHR, 0.63 [95% CI 0.57–0.70]). Among female patients, MPH users had a significantly lower risk of PP than non-MPH users (aHR, 0.51 [95% CI 0.44–0.59]). However, among male patients, MPH users showed only a trend toward a lower risk of PP compared with non-MPH users (aHR, 0.91 [95% CI 0.79–1.12]). The mean and standard deviation of days from the prescription of MPH to diagnosis of PP were 1094.29 days and 668.06 days, respectively. The minimum duration is 99 days. In the moderation analysis of sex, we also found a negative effect modification by female gender on the association between MPH and PP among ADHD patients, both additive (RERI, −4.38 [95% CI −5.51, −3.25]) and multiplicative (aHR, 0.54 [95% CI 0.43, 0.67]).

## 4. Discussion

Our study found that patients with ADHD were associated with a greater risk of PP. The association between ADHD and PP was more prominent in girls from the perspective of the additive scale. The additive scale is also a more relevant measure when assessing the public health significance of effect modification [35]. Comorbid tics or intellectual disability weakened the association between ADHD and PP on both the additive and multiplicative scales. Among patients with ADHD, MPH users were associated with a lower risk of PP, especially in girls.

Our findings suggested that patients with ADHD are vulnerable to PP, which is consistent with previous findings by Pai et al. [2]. This finding is also supported by animal studies [39,40,41]. The activation of the dopamine D1 receptor (D1R) and inhibition of the dopamine D2 receptor (D2R) were found to be responsible for ADHD-like behaviors in mice [39]. The activation of D1R stimulated GnRH release, and D2R served as a brake on the GnRH release [41,42]. Persistent GnRH release would initiate puberty [40]. Thus, an imbalance between D1R and D2R in patients with ADHD may contribute to PP susceptibility.

Another proposed mechanism is disruption in the immune system and chronic inflammation in patients with ADHD [43,44]. Chronic inflammation is one of the causes of hypothalamic–pituitary–adrenal axis (HPA) dysfunction. The alteration of the HPA axis related to ADHD symptoms are reported in the literature [45,46]. The attenuated activation of the HPA was linked to PP in one study [47]. Its authors inferred that this might have resulted from the suppression of the hypothalamic–pituitary–gonadal axis by the HPA axis. On the contrary, several studies reported no association or negative association between ADHD and inflammation or HPA-axis dysregulation [48,49]. Vogel et al. found the association was driven by comorbidity, such as depression and anxiety, rather than ADHD [49]. There are also studies indicating the link between obesity-induced hypothalamic inflammation and PP [50]. Emerging research and a recent meta-analysis have shown a significant association between obesity and ADHD in both adults and children [51]. However, the causal relationship between ADHD, obesity, and PP remains elusive. Furthermore, a recent study identified two inflammatory biotypes in ADHD, that is, high and low inflammatory statuses [52]. These two distinct biotypes might help explain the mixed results of the association between ADHD and inflammation.

Our study further pointed out that the association between ADHD and PP varies across sexes. The effect of ADHD on PP was significantly greater in females than in males, with an RERI of 8.56, although the multiplicative moderating effect was null. This negative-additive–null-multiplicative effect modification could be explained by the great disparity in baseline PP risk between males and females (1.69% and 0.18%, respectively) [35]. On the other hand, an RERI of this magnitude suggests that there were cases in our study population where PP would occur only if both criteria (i.e., female and ADHD) were met but would not occur if only one criterion (i.e., female only or ADHD only) was met [35]. Therefore, girls with ADHD may possess a unique pathology mechanism for PP. This phenomenon could be supported by the sexual dimorphism of hypothalamic D2R levels [53]. During prepuberty and puberty, D2R levels decline in both sexes, but this occurs more rapidly in females [53]. As mentioned above, a decrease in D2R loosens the brake on GnRH and initiates puberty, which may make girls with ADHD more susceptible to PP.

ADHD comorbid with different neuropsychiatric disorders may moderate the association between ADHD and PP. Both tics and intellectual disability existed negative-additive–negative-multiplicative effect modifications. Autistic spectrum disorder and obsessive–compulsive disorder only existed null-additive–negative-multiplicative effect modifications. Additive effect modification is a more relevant measure for public health and may indicate possible biological independence [35]. Thus, we adopted a conservative approach to explain the moderating effect of comorbidities; that is, the effect of ADHD on PP was attenuated in subgroups comorbid with tics or intellectual disability. The possible underlying mechanism may be explained by the cross-antagonistic interaction between the histamine H3 receptor (H3R) and D1R [54]. H3R mediated the braking of D1R signaling [55]. In animal models, the activation of H3R has been proposed to be linked with tic-like behaviors and intellectual disability [56,57]. Thus, the activation of H3R inhibited the onset of puberty by inactivating D1R signaling, which would antagonize the effects of ADHD on PP.

MPH treatment in patients with ADHD appeared to be protective against PP. In addition, among them, MPH was more beneficial for girls than for boys. This phenomenon can be partially explained by previous animal studies. According to previous rodent studies, MPH itself, in the absence of ADHD, could alter the timing of puberty [23,24,25,26]. Chatterjee-Chakrabarty et al. [23], Guarraci et al. [25], and Khoubbieh et al. [24] reported that the administration of MPH in the early life of female mice delayed puberty. Khoubbieh et al. reported that MPH use accelerated puberty in male mice [24]. However, in one primate study, the administration of MPH delayed puberty in male rhesus monkeys [26]. Overall, the effects of MPH on females were consistent and manifested as delayed puberty, but the effects on males were still conflictual. More research is warranted to explore the complex biological interactions between MPH and sex-specific puberty.

This study has some limitations. First, because of the nature of the database, we cannot rule out some important confounding effects, for example, environmental chemical exposure [58,59], prenatal and perinatal events [60,61], and food and nutrient exposure [62,63,64]. As mentioned, the NHIRD did not contain the data regarding inflammatory markers, body mass index, or records of life stress events. We were unable to verify the inflammatory hypothesis moderating association between ADHD and PP. Second, according to the ICD systems, we cannot distinguish peripheral PP from central PP. Third, we included some comorbidities, such as autistic spectrum disorder, in our analysis to avoid confounding effects. However, comorbidities that manifest and are diagnosed later in life would not be detected in the medical-claims data during the follow-up period and, therefore, were not analyzed in this study. Fourth, premature thelarche and premature adrenarche were not able to be differentiated from PP, which could have led to overestimations of the risk of PP. In addition, identifying cases with ADHD based solely on primary medical records may lead to overestimations of the prevalence of ADHD. This study may have encountered misclassifications of MPH, as patients prescribed MPH may not have actually taken the medication, leading to incorrect categorizations as MPH users. In this case, there is no reason to assume that the decision to take MPH is influenced by the risk of PP. Such nondifferential misclassification [35], meaning that the misclassification is unrelated to the outcome, would bias the results toward a null hypothesis [35]. Thus, even if misclassification exists in this study, it would not change the direction of the association between exposure and outcome [35]. Finally, due to the observational nature of this study, we cannot establish an exact causal relationship between MPH and the risk of PP. There may be unmeasured confounding factors or other upstream factors affecting the association between MPH and PP. For instance, specific ADHD phenotypes or severity levels might drive the prescription of MPH, or MPH could act as a proxy variable for some unknown factor. Therefore, the potential protective effect of MPH on PP must be interpreted with caution.

## 5. Conclusions

Our study found that children with ADHD were at a greater risk of PP, and girls with ADHD were a particularly vulnerable group. Comorbidity with tics or intellectual disability was associated with a lower risk of PP. MPH appeared to be protective against PP in patients with ADHD, especially in girls. However, these preliminary results need further validation. This study provided an important basis for understanding the relationship between ADHD, MPH, and PP. The results highlight the need for clinicians to closely monitor the timing of puberty in children with ADHD, particularly girls, to mitigate the challenges associated with early pubertal onset. Further research, including both animal and human studies, is essential to investigate the impact of MPH on pubertal development. Such studies could clarify the mechanisms by which MPH influences growth and pubertal development, potentially enabling more targeted interventions for children with ADHD. Additionally, exploring the effects of ADHD-related comorbidities on puberty can enhance our understanding of pubertal developmental trajectories in this population.

## Figures and Tables

**Table 1:** Demographic characteristics of ADHD and non-ADHD individuals.

Characteristics	ADHD n = 186,681	Non-ADHD n = 3,155,396
Age, mean (S.D.) years	14.8 (3.8)	15.2 (4.0)
Sex, n (%)		
Male, n (%)	146,006 (78.21)	1,595,547 (50.57)
Female, n (%)	40,675 (21.79)	1,559,849 (49.43)
Low income, n (%)	21,687 (11.62)	302,099 (9.57)
Psychiatric comorbidity		
ASD, n (%)	10,519 (5.63)	18,801 (0.60)
Tics, n (%)	11,111 (5.95)	14,354 (0.45)
OCD, n (%)	1429 (0.77)	3468 (0.11)
Intellectual disability, n (%)	18,112 (9.70)	15,037 (0.48)
Anxiety, n (%)	8173 (4.38)	19,449 (0.61)
Epilepsy, n (%)	4529 (2.43)	12,175 (0.39)
MPH prescription, n (%)	122,302 (65.51)	-
PP, n (%)	2042 (1.09)	29,299 (0.93)

Abbreviations: ADHD: attention-deficit/hyperactivity disorder; ASD: autistic spectrum disorder; OCD: obsessive–compulsive disorder; n: number; S.D.: standard deviation; PP: precocious puberty.

**Table 2:** Cox proportional hazard regression model analysis for the risk of precocious puberty between the ADHD and non-ADHD group and effect modification by sex and different neuropsychiatric comorbidities.

Group		Non-ADHD Subgroup		ADHD Subgroup		aHRs (95% CI) within Strata of Effect Modifiers ^a^	RERI (95% CI) Effect Modification Based on Additive Scale	aHR (95% CI) Effect Modification Based on Multiplicative Scale ^a^
		n (PP/Non-ADHD) (%)	aHR (95% CI)	n (PP/ADHD) (%)	aHR (95% CI)			
ADHD		2042/186,681 (1.09)	1.00	29,299/3,155,396 (0.93)	**2.01** (1.91–2.11) *	-	-	-
Moderation effect of sex								
Male		2887/1,595,547 (0.18)	1.00	600/146,006 (0.41)	2.01 (1.83, 2.20)	**2.05** (1.86–2.26) *	-	-
Female		26,412/1,559,849 (1.69)	9.52 (9.16, 9.89)	1442/40,675 (3.55)	19.09 (17.88, 20.38)	**1.99** (1.88–2.11) *	**8.56** (7.48, 9.64) *	1.00 (0.90, 1.11)
Moderation effect of neuropsychiatric comorbidities								
ASD	No	29,168/3,144,877 (0.93)	1.00	1820/167,880 (1.08)	2.05 (1.96, 2.16)	**2.06** (1.96–2.16) *		
	Yes	131/10,519 (1.25)	2.58 (2.17, 3.07)	222/18,801 (1.18)	3.08 (2.69, 3.53)	1.21 (0.97–1.51)	−0.56 (−1.17, 0.05)	**0.58** (0.46, 0.72) *
Tics	No	29,113/3,141,042 (0.93)	1.00	1956/175,570 (1.11)	2.06 (1.96, 2.17)	**2.06** (1.96–2.16) *		
	Yes	186/14,354 (1.30)	2.20 (1.91, 2.55)	86/11,111 (0.77)	2.02 (1.64, 2.51)	0.97 (0.74–1.28)	**−1.24** (−1.78, −0.70) *	**0.45** (0.34, 0.58) *
Intellectual disability	No	29,149/3,140,359 (0.93)	1.00	1843/168,569 (1.09)	2.06 (1.95, 2.16)	**2.05** (1.95–2.16) *		
	Yes	158/15,037 (1.05)	1.28 (1.10, 1.50)	199/18,112 (1.10)	1.30 (1.05, 1.61)	**1.64** (1.42–1.89) *	**−0.70** (−1.02, −0.38) *	**0.62** (0.50, 0.77) *
Anxiety	No	28,975/3,135,947 (0.92)	1.00	1917/178,508 (1.07)	2.02 (1.92, 2.13)	**2.03** (1.93–2.13) *		
	Yes	324/19,449 (1.67)	1.32 (1.17, 1.48)	125/8173 (1.53)	1.62 (1.29, 2.04)	**2.17** (1.81–2.60) *	−0.18 (−0.60, 0.24)	0.81 (0.66, 1.00)
OCD	No	29,226/3,151,928 (0.93)	1.00	2016/185,252 (1.09)	2.02 (1.92, 2.12)	**2.02** (1.92–2.12) *		
	Yes	73/3468 (2.10)	1.70 (1.34, 2.17)	26/1429 (1.82)	1.98 (1.34, 2.94)	1.09 (0.66–1.79)	−0.74 (−1.60, 0.12)	**0.58** (0.37, 0.90) *
Epilepsy	No	29,156/3,143,221 (0.93)	1.00	1968/182,152 (1.08)	2.01 (1.91, 2.11)	**2.02** (1.92–2.12) *		
	Yes	143/12,175 (1.17)	1.31 (1.11, 1.55)	74/4529 (1.63)	2.35 (1.86, 2.97)	**1.16** (1.18–2.20) *	0.03 (−0.56, 0.62)	0.89 (0.67, 1.19)

Abbreviations: ADHD: attention-deficit/hyperactivity disorder; aHR: adjusted hazard ratio; ASD: autistic spectrum disorder; CI: confidence interval; OCD: obsessive–compulsive disorder; n: number; PP: precocious puberty; RERI: relative excess risk due to interaction. Bold values and * denote statistical significance. ^a^ Adjusted model controlled for sex, low-income households, and neuropsychiatric comorbidities (if these covariates were not specified as effect modifiers)

**Table 3:** Cox proportional hazard regression model analysis of MPH use on the risk of precocious puberty among patients with ADHD and effect modification by sex.

Group	ADHD without MPH		ADHD with MPH		aHRs (95% CI) within Strata of Effect Modifier ^a^	RERI (95% CI) Effect Modification Based on Additive Scale	aHR (95% CI) Effect Modification Based on Multiplicative Scale ^a^
	n (PP/Non-MPH Users) (%)	aHR (95% CI)	n (PP/MPH Users) (%)	aHR (95% CI)			
MPH	841/63,767 (1.32)	1.00	590/122,302 (0.48)	**0.63** (0.57–0.70) *	-	-	-
Moderation effect of sex							
Male	230/47,004 (0.49)	1.00	292/98,923 (0.30)	0.94 (0.79–1.12)	0.91 (0.77–1.08)	-	-
Female	611/16,763 (3.64)	8.97 (7.70, 10.45)	298/23,379 (1.27)	4.53 (3.80–5.39)	**0.51** (0.44–0.59) *	**−4.38** (−5.51, −3.25) *	**0.54** (0.43, 0.67) *

Abbreviations: ADHD: attention-deficit/hyperactivity disorder; aHR: adjusted hazard ratio; CI: confidence interval; n: number; MPH: methylphenidate; PP: precocious puberty; RERI: relative excess risk due to interaction. Bold values and * denote statistical significance. ^a^ Adjusted model controlled for sex, low-income households, and neuropsychiatric comorbidities (if these covariates were not specified as effect modifiers).

## Data Availability

This study was based on the National Health Insurance Research Database, managed by the Health and Welfare Data Science Center. This database is owned by the Ministry of Health and Welfare (Taiwan).

## Supplementary Materials

The following supporting information can be downloaded at https://www.mdpi.com/article/10.3390/biomedicines12102304/s1, Table S1: International Classification of Diseases, Ninth Revision, Clinical Modification (ICD9-CM) and International Classification of Diseases, Ninth Revision, Clinical Modification (ICD-10) for diseases of exclusion criteria; Table S2: International Classification of Diseases, Ninth Revision, Clinical Modification (ICD9-CM) and International Classification of Diseases, Ninth Revision, Clinical Modification (ICD-10) for diseases of the neuropsychiatric comorbidities; Table S3: Prescription drug codes for methylphenidate from the National Health Insurance Research Database (NHIRD).
