# Supplementary material for: ADHD and Risk of Precocious Puberty: Considering the Impact of MPH
Source: Biomedicines. 2024 Oct 10;12(10):2304. doi: 10.3390/biomedicines12102304 (PMC11505504; doi:10.3390/biomedicines12102304)
Supplement: Supplementary file 1 [file biomedicines-12-02304-s001.zip › biomedicines-3210660-supplementary.pdf]

Supplementary Materials

ADHD and Risk of Precocious Puberty: Considering the Impact of MPH

Methods

Table S1. International Classification of Diseases, Ninth Revision, Clinical Modification (ICD9-CM) and International Classification of Diseases, Ninth Revision, Clinical Modification (ICD-10) for diseases of exclusion criteria

Table S2. International Classification of Diseases, Ninth Revision, Clinical Modification (ICD9-CM) and International Classification of Diseases, Ninth Revision, Clinical Modification (ICD-10) for diseases of the neuropsychiatric comorbidities

Table S3. Prescription drug codes for methylphenidate from the National Health Insurance Research Database (NHIRD)

Table S1. International Classification of Diseases, Ninth Revision, Clinical Modification (ICD9-CM) and International Classification of Diseases, Ninth Revision, Clinical Modification (ICD-10) for diseases of exclusion criteria.

Diseases	ICD-9CM	ICD-10
Primary hypothyroidism	244.9	E03.9
Central nervous system infection	320, 321, 322, 323, 324, 325, 326, 327	G00, G01, G02, G03, G04, G05, G06, G07
Congenital abnormalities of the central nervous system	741, 742	Q05, Q07
Septo-optic dysplasia	744	Q04
Tuberous sclerosis	759.5	Q85.1
Sturge-Weber syndrome	759.6	Q85.8, Q85.9
Ever radiation to the central nervous system	V58.0	Z51.0

Table S2. International Classification of Diseases, Ninth Revision, Clinical Modification (ICD9-CM) and International Classification of Diseases, Ninth Revision, Clinical Modification (ICD-10) for diseases of the neuropsychiatric comorbidities

Diseases	ICD-9CM	ICD-10
ASD	299	F84
Tics	307.2	F95
OCD	300.3	F42
Anxiety	300.0	F41.0, F41.1, F41.9
Intellectual disability	317, 318, 319	F70, F71, F72, F73, F78, F79
Epilepsy	345	G40, G41

ASD autistic spectrum disorder; *OCD* obsessive–compulsive disorder

Table S3. Prescription drug codes for methylphenidate from the National Health Insurance Research Database (NHIRD).

A030740100	A037906100	AC30740100	AC59242100
AC59417100	AC59744100	AC59797100	AC60152100
AC60153100	AC60165100	B023731100	B023880100
B023999100	B024229100	B024979100	B025016100
B025332100	B025333100	B025334100	B025335100
BC23731100	BC23880100	BC23999100	BC24229100
BC24979100	BC25016100	BC25332100	BC25334100
BC25335100	BC26571100	BC27080100	
